# Estrogen receptor (ESR) 1 promotes uterine leiomyoma cell proliferation by enhancing mitochondrial energy metabolism via Wnt/β-catenin pathway activation

**DOI:** 10.1186/s12905-025-04256-3

**Published:** 2026-01-13

**Authors:** Qi Wu, Suning Bai, Xiaoyan Duan, Liyun Song, Lina Han, Qing Guo

**Affiliations:** 1 Hebei Medical University, Shijiazhuang, 050000 China; 2 Department of Gynecology, Hebei General Hospital, Shijiazhuang, 050000 China; 3 Department of Obstetrics and Gynecology, The Fourth Hospital of Shijiazhuang, 16 Tangu North Street, Chang’an District, Shijiazhuang, Hebei Province 050000 China

**Keywords:** Uterine leiomyoma, ESR1, Wnt/β-catenin signaling pathway, Mitochondrial energy metabolism, Cell proliferation

## Abstract

**Background:**

Hyperactive mitochondrial energy metabolism supports the rapid proliferation of uterine leiomyoma cells. Estrogen receptor (ESR)-1 is associated with mitochondrial functions and the Wnt/β-catenin pathway; however, its regulatory roles in this pathway remain unclear. Therefore, we aimed to assess whether ESR1 enhances mitochondrial energy metabolism and regulates ferroptosis by activating the Wnt/β-catenin pathway.

**Methods:**

Using the GSE593 dataset from the Gene Expression Omnibus and GeneCards databases, we identified *ESR1* as the key hub gene. *ESR1* knockdown rat uterine leiomyoma cell model (ELT3) was constructed to evaluate the cell proliferation, ferroptosis, and mitochondrial function changes. Moreover, Wnt/β-catenin pathway activator 1, AG1 was used to verify the regulatory relationship between ESR1 and this pathway.

**Results:**

Bioinformatics analysis identified 22 mitochondria-related differentially expressed genes. Among these, ESR1 exhibited the highest connectivity in the protein–protein interaction network (degree = 5). ESR1 was highly expressed in ELT3 cells. *ESR1* knockdown significantly inhibited cell proliferation, induced ferroptosis (increased the Fe²⁺ and malondialdehyde levels and caused disappearance of mitochondrial cristae), and reduced the mitochondrial membrane potential and levels of key energy metabolism-related proteins. Mechanistically, *ESR1* knockdown inhibited the Wnt/β-catenin pathway (downregulated the β-catenin and p-GSK-3β(Ser9)/GSK3β levels). However, AG1 reversed these effects, restoring cell proliferation and mitochondrial functions.

**Conclusions:**

Overall, ESR1 enhanced mitochondrial oxidative phosphorylation by activating the Wnt/β-catenin signaling pathway, driving uterine leiomyoma cell proliferation. However, our findings suggest that its knockdown induces ferroptosis in leiomyoma cells, revealing a new strategy to prevent uterine leiomyoma recurrence.

**Supplementary Information:**

The online version contains supplementary material available at 10.1186/s12905-025-04256-3.

## Background

Uterine leiomyoma is the most common benign tumor of the female reproductive system, with an incidence rate of 20–40% among women of reproductive age [1]. Its existing clinical treatment strategies exhibit various limitations. Drug treatment mainly involves gonadotropin-releasing hormone agonists and progesterone antagonists. However, its recurrence rate is high post-drug withdrawal, and long-term drug use exerts various side effects [2]. Complete removal of diffuse lesions during surgical treatment remains difficult, increasing the risk of uterine rupture during pregnancy. Existing treatments do not target the core metabolic mechanisms of leiomyoma, only focusing on symptom control [3]. Mitochondrial energy metabolism is potentially the core driving force for abnormal leiomyoma cell proliferation. Leiomyoma cells exhibit various characteristics, such as increased mitochondrial numbers, enhanced oxidative phosphorylation (OXPHOS), and hyperactive ATP synthesis. This “over-functioning” of energy metabolism is crucial to meet the exuberant demands of abnormal rapid proliferation, hypertrophy of leiomyoma cells, and excessive extracellular matrix deposition by providing a large amount of biological energy (ATP) and biosynthetic precursors, thereby becoming the “energy engine” for maintaining leiomyoma growth [4–6]. Therefore, targeting pathways related to mitochondrial energy metabolism is a potential strategy to overcome the current treatment bottlenecks.

Estrogen receptor (ESR)-1, a nuclear receptor superfamily member, performs other functions beyond its classical genomic functions. Through non-genomic signaling pathways, it is deeply involved in the regulation of cellular energy metabolism homeostasis and closely associated with mitochondrial functions. Multiple signaling networks regulate the biosynthesis, respiratory chain activity, and ATP production in mitochondria, known as the energy factories of cells. ESR1 affects the mitochondria-related gene (e.g., optic atrophy 1 and mitofusin 1) expression and respiratory enzyme activity, thereby directly or indirectly regulating the OXPHOS efficiency [7, 8]. Notably, ESR1 exhibits a close crosstalk with the Wnt/β-catenin signaling pathway. ESR1 activation significantly increases β-catenin expression [9]. Upon entering the nucleus, β-catenin, a core effector molecule of the classical Wnt pathway, activates the target genes, such as c-Myc and cyclin D1 [10], promoting cell proliferation and mitochondrial OXPHOS by increasing the expression levels of metabolic enzymes, such as pyruvate dehydrogenase kinase [11]. ERalpha and the Wnt/β-catenin pathway are crucial for the progression of most endometrial cancers [9]. The crosstalk between ESR1 and β-catenin is considered one of the key mechanisms driving tumor progression, and β-catenin can act as a transcriptional co-activator of ER [12]. Additionally, in the normal cyclic regeneration of the endometrium and the regulation of ovarian function, these two pathways also show a synergistic effect [13]. Collectively, these studies suggest that the relationship between ESR1 and Wnt/β-catenin has also been reported in other female reproductive system diseases. These reports suggest that the mechanisms by which the ESR1–Wnt/β-catenin signaling axis coordinately regulates mitochondrial energy metabolism show significant potential for targeted interventions.

In this study, we identified the differentially expressed genes (DEGs) between uterine fibroids and healthy tissues using the GSE593 dataset. Potential therapeutic targets for uterine fibroids were explored via weighted gene co-expression network analysis (WGCNA), and a gene set related to mitochondrial diseases (1,518 genes) was obtained from the GeneCards database. Using a Venn diagram, 22 candidate genes were identified at the intersection of the three gene sets. Candidate genes with connectivity ≥ 3, namely *ESR1* (node degree = 5), peroxisome proliferator-activated receptor gamma (*PPARG*; node degree = 3), and glucokinase (*GCK*; node degree = 3), were screened using the protein–protein interaction (PPI) network. Our experiments revealed that ESR1 was abnormally expressed in the uterine leiomyoma cells and exhibited the highest node degree in the PPI network. Therefore, we focused on ESR1 in subsequent analyses. Considering its association with the Wnt/β-catenin signaling pathway, we hypothesized that ESR1 influences mitochondrial energy metabolism in uterine leiomyoma cells, affecting their proliferation by mediating the Wnt/β-catenin signaling pathway. To verify this, we investigated the mitochondrial energy metabolism and cell proliferation in an *ESR1* knockdown cell model. Mechanistically, we used the Wnt/β-catenin agonist 1 (AG1, Wnt/β-catenin signaling pathway activator) to assess the relationship between ESR1 and the Wnt/β-catenin signaling pathway and identify novel therapeutic targets for uterine leiomyoma.

## Methods

### DEG analysis

The GSE593 dataset, titled “Uterine Fibroid and Normal Myometrial Expression Profiles-U133 Arrays.” was downloaded from the Gene Expression Omnibus database. This dataset originated from the School of Medicine at the University of California, Davis. It encompasses gene expression data from five uterine fibroid and five normal myometrial tissue samples. After retrieving the data using the GeoChina function, an expression matrix (Exprs) was extracted and subjected to quality control. Raw data were log-transformed (log2(x + 1)) to satisfy the assumption of a normal distribution. A linear model from the limma package was used for differential analysis. The thresholds were set at |logFC| > 1 and *P* < 0.05. To visually represent the data characteristics, “ggplot2” [14] and “ComplexHeatmap” [15] software packages were used to generate the volcano plots and heatmaps, respectively.

### Screening for mitochondrial disease-related genes

Top 40% of genes showing the greatest fluctuations were selected to construct a gene co-expression network. A soft threshold (power = 3) was used to build an adjacency matrix, from which a topological overlap matrix was generated. Using the dynamic tree-cutting algorithm, eight modules were identified, with each module corresponding to a specific biological function. Correlation between the module eigengene and phenotype (disease status) was assessed; “brown” module was significantly positively correlated with the disease group. Through the correlation analysis of the module membership SHIP and gene significance, 553 core differential genes within the “brown” module were screened out. Using the advanced search module of the GeneCards database (https://www.genecards.org/), with relevance score > 10 (comprehensive GeneCards score integrating evidence weights, such as expression, pathways, and interactions), 1,518 mitochondrial-related genes were identified. DEGs, differential genes from WGCNA, and mitochondria-related genes were used to draw a Venn diagram using the Venn Diagram package [16], and 22 intersecting genes were obtained. These 22 genes were subsequently imported into STRING 12.0 (https://cn.string-db.org) to construct a PPI network.

### Cell sources and culture

ELT3 rat uterine leiomyoma cell line was purchased from the American Type Culture Collection. Rat uterine smooth muscle cells (UtSMCs) were obtained from Qingqi (Shanghai) Biotechnology Development Co., Ltd. All cells were identified via short tandem repeat typing. The results confirmed the identity of the cells and revealed no cross- or mycoplasma contamination. The cells were cultured in the 1640 medium (C0893; Shanghai Biyuntian Biotechnology Co., Ltd., China) supplemented with 10% fetal bovine serum (40131ES76; Yeasen Biotechnology (Shanghai) Co., Ltd., China) and 1% penicillin–streptomycin double-antibody solution (60290ES; Yeasen Biotechnology (Shanghai) Co., Ltd.). The culture was maintained in a constant temperature and humidity incubator (51023126; Thermo Fisher Scientific, China) at 37 °C and 5% CO₂. The cells were passaged regularly to maintain the exponential growth phase. Cells in the logarithmic growth phase with approximately 70–80% confluence were selected for subsequent experiments.

### Cell transfection and experimental groups

ELT3 cells and UtSMCs in the exponential growth phase were collected. After adding a trypsin solution (3 mL; 40126ES; Yeasen Biotechnology (Shanghai) Co., Ltd.), the cells were incubated for 3 min. Next, 5 mL of the 1640 medium was added to collect the cell suspension in a centrifuge tube, which was centrifuged at 1300 × *g* for 3 min. The used culture medium was removed, and the cells were resuspended in 3 mL of fresh medium. Cell concentration was then determined using a cell counter (0267012; Thermo Fisher Scientific). Cells were plated in six-well plates at a concentration of 1 × 10^5^ cells per well. The plates were then incubated overnight. Small interfering RNA (siRNA) sequences specifically designed to knock down *ESR1* were prepared. According to the instructions of the Lip 3000 Transfection Kit (L3000075; Thermo Fisher Scientific), siRNAs and transfection reagent were mixed and added to the cells, followed by incubation for 4 h. After incubation, the medium was replaced with a fresh medium, and the cells were incubated for an additional 48 h. Subsequently, transfection efficiency was determined via reverse transcription-quantitative polymerase chain reaction (RT-qPCR). The siRNA interference sequences and negative control groups are presented in Supplementary Table 1.

For the *ESR1* knockdown model, three groups were established: SiNC (transfected with the negative control siRNA) and siESR1-1 and siESR1-2 (transfected with the ESR1-targeted siRNA sequences) groups. AG1, a Wnt/β-catenin signaling pathway activator, was used in this study. To assess the functional association between ESR1 and the Wnt/β-catenin pathway, additional pathway activation intervention (siNC. siESR1-1, and siESR1-1 + AG1) groups treated with 5 µM AG1 (HY-114321; MedChemExpress, China) were established [17]. AG1 was added 24 h after transfection, and the cells were continuously treated for 48 h.

### Cell counting kit (CCK)-8 assay

ELT3 cells in the exponential phase of growth were plated at a density of 3500 cells per well in a 96-well plate and allowed to adhere overnight under standard culture conditions. Subsequently, the cells were treated according to each group requirements described above. After treatment, 10 µL of CCK8 solution (40203ES; Yeasen Biotechnology (Shanghai) Co., Ltd.) was added to each well, and incubation was continued for 3 h. After incubation, the 96-well plate was placed in a microplate reader (A51119700DPC; Thermo Fisher Scientific), and absorbance was measured at a wavelength of 450 nm.

#### 5-ethynyl-2’-deoxyuridine (EdU) staining

ELT3 cells under good growth conditions were seeded in a 24-well plate (with cover slips) at a density of 5 × 10^3^cells/well and incubated overnight. The old culture medium was aspirated, and a fresh complete medium containing 10 µmol/L EdU (40275ES; Yeasen Biotechnology (Shanghai) Co., Ltd.) was added. Incubation was continued for 2 h. After aspirating the EdU-containing medium, 4% paraformaldehyde was added, and the cells were incubated for 15 min. Next, 0.5% Triton X-100 was added, and the cells were incubated for 20 min. According to the kit instructions, Click reaction solution was prepared, and 200 µL of the solution was added to each well. The reaction was performed in the dark for 30 min. Then, phosphate-buffered saline containing 4′,6-diamidino-2-phenylindole dihydrochloride (1 µg/mL) was added to stain the nuclei for 10 min in the dark. Images were obtained using a confocal microscope (KS-X1500; KathMatic, China). Finally, EdU-positive cell proportion was calculated using the ImageJ 1.5.2a software.

#### 5,5’,6,6’-tetrachloro-1,1’,3,3’-tetraethylbenzimidazolcarbocyanine iodide (JC-1) fluorescent probe

ELT3 cells in the logarithmic growth phase were plated in six-well plates at a density of 1.5 × 10^5^ cells per well and maintained at 37 °C under 5% CO₂ for 24 h to allow for complete adhesion. Following treatment according to the respective experimental group protocols, cells were incubated with JC-1 working solution at a final concentration of 10 µg/mL (40706ES, Yeasen Biotechnology (Shanghai) Co., Ltd.) to assess mitochondrial membrane potential. The cells were incubated at 37 °C in the dark for 20 min. Subsequently, JC-1 staining solution was aspirated, and the cells were washed twice with phosphate-buffered saline. Images were obtained via confocal microscopy. The ratio of red to green fluorescence intensity was calculated using the ImageJ 1.5.2a software.

### Mitochondrial structure analysis via transmission electron microscopy

ELT3 cells in the logarithmic growth phase were seeded in a 6-well plate (with coverslips) at a density of 5 × 10^5^ cells/well and cultured for 24 h until they reached 80% confluence. The culture medium was aspirated, and pre-cooled 2.5% glutaraldehyde was quickly added. The cells were fixed at 4 °C for 2 h, transferred to an Eppendorf tube, and fixed overnight at 4 °C. Then, 1% osmium tetroxide solution was added, and the cells were fixed at 4 °C in the dark for 1.5 h. After dehydrating the cells using an ethanol gradient, acetone was then added, and the mixture was incubated for 10 min. Coverslips were placed on an embedding plate, and fresh resin was poured in the marked direction. Then, 70-nm sections were cut using an ultramicrotome (Ultra 45°; Daitome, China). The sections were stained with uranyl acetate at 25 °C for 20 min and subsequently with lead citrate in the dark for 5 min. Finally, changes in the cell mitochondrial structure in each group were observed using a transmission electron microscope (HT7800; Hitachi, China).

### Determination of Fe²⁺ and malondialdehyde (MDA) levels

ELT3 cells in the logarithmic growth phase were seeded in a six-well plate at a density of 3 × 10^5^ cells/well and incubated overnight. The cells were treated according to the requirements of each group to obtain the cell pellets. The samples were processed following the procedures of the Fe²⁺ (BC1955; Beijing Solarbio Science & Technology Co., Ltd., China) and MDA (J23789; Wuhan Jilid Biotechnology Co., Ltd.) detection kits. After processing, optical density of Fe²⁺ was measured at 593 nm, whereas that of MDA was measured at 450 nm. The data were used for statistical analyses.

### RT-qPCR

After treating the cells in each group as described above, the cell suspensions were collected and centrifuged at 1200 × *g* for 3 min to obtain the cell pellets. Total cellular RNA was extracted using the TRIzol kit (R0016; Shanghai Biyuntian Biotechnology Co., Ltd.). Add Deoxyribonuclease I (D7073, Beyotime Biotechnology, China) and incubate at 37 °C for 30 min. RNA concentration and purity were measured using a spectrophotometer, and cDNA was synthesized using a reverse transcription kit (R0016; Shanghai Biyuntian Biotechnology Co., Ltd.) through a 60-min reaction at 42 °C. Subsequently, a qPCR system, including the SYBR Green Master Mix (D7501; Shanghai Biyuntian Biotechnology Co., Ltd.), gene-specific forward and reverse primers, diluted cDNA template, and nuclease-free water, was prepared. The reaction mixture was added to a 96-well plate and placed in the real-time fluorescence quantitative PCR instrument (4351405; Thermo Fisher Scientific, USA) for amplification. The program was set as follows: Pre-denaturation at 95 °C for 10 min, followed by 40 cycles of 95 °C for 15 s, 60 °C for 15 s, and 95 °C for 15 s. The data were saved. Finally, data analysis was performed using the 2^⁻ΔΔCt^ method. Glyceraldehyde-3-phosphate dehydrogenase was used as an internal reference to normalize the target gene levels [18]. All primers are listed in Supplementary Table 2. Each experimental condition included 3 technical replicates, and all experiments were independently repeated 3 times (i.e., *n* = 3 biological replicates).

### Western blotting

After treating the cells in each group as described above, the cell suspensions were collected and centrifuged at 1200 × *g* for 3 min to obtain the cell pellets. RAPI (P0013C; Shanghai Biyuntian Biotechnology Co., Ltd.) was added, and the mixture was incubated on ice for 30 min. Protein concentration was determined using the BCA protein quantification kit (P0012S; Shanghai Biyuntian Biotechnology Co., Ltd.). Protein loading buffer (P0015A; Shanghai Biyuntian Biotechnology Co., Ltd.) was added to adjust the protein concentration in each group for consistency. Then, protein solution was incubated in a 95 °C metal bath (E1525; Shanghai Biyuntian Biotechnology Co., Ltd.) for 15 min. After natural cooling, 30 µg of protein solution per well was added to the electrophoresis tank, and constant-voltage mode (120 V) was set and run for 90 min. The gel was transferred to a polyvinylidene difluoride (PVDF) membrane in cross-flow mode (260 mA) for 60 min. The PVDF membrane was placed in skim milk powder (P0216; Shanghai Biyuntian Biotechnology Co., Ltd.) and incubated at 25 °C for 2 h. After adding primary antibodies, the membrane was incubated overnight. Following three 30 min washes with Tris-buffered saline containing Tween-20 (TBST), the membrane was incubated with the corresponding secondary antibodies at 25 °C for 2 h. The membrane was again washed thrice with Tris-buffered saline containing Tween-20 for 5 min each, a chemiluminescent hypersensitive solution (P0018FM; Shanghai Biyuntian Biotechnology Co., Ltd.) was added, and the membrane was incubated for 20 s. The PVDF membrane was visualized using a chemiluminescence gel imaging system (A44114; Thermo Fisher Scientific), and the original images were retained for subsequent analysis. Gray values of the bands were calculated using the Image J 1.5.2a software. The relative target gene expression level was indicated by the ratio of the gray value of the target gene to that of the internal reference gene, glyceraldehyde-3-phosphate dehydrogenase. All antibody information and dilution ratios are presented in Supplementary Table 3. Full, uncropped western blot images can be found in the Supplementary Materials.

### Statistical analyses

All statistical analyses were performed using GraphPad Prism (9.5.0), with data from a minimum of three independent biological replicates expressed as mean ± standard deviation. We employed an unpaired two-tailed Student’s t-test for comparisons between two groups and a one-way analysis of variance (ANOVA) for comparisons across multiple groups. When analysis of variance indicated a significant difference (*P* < 0.05), Tukey’s post-hoc test was used for multiple comparisons.

## Results

### Differential gene analysis of the GSE593 dataset

**Fig. 1:**
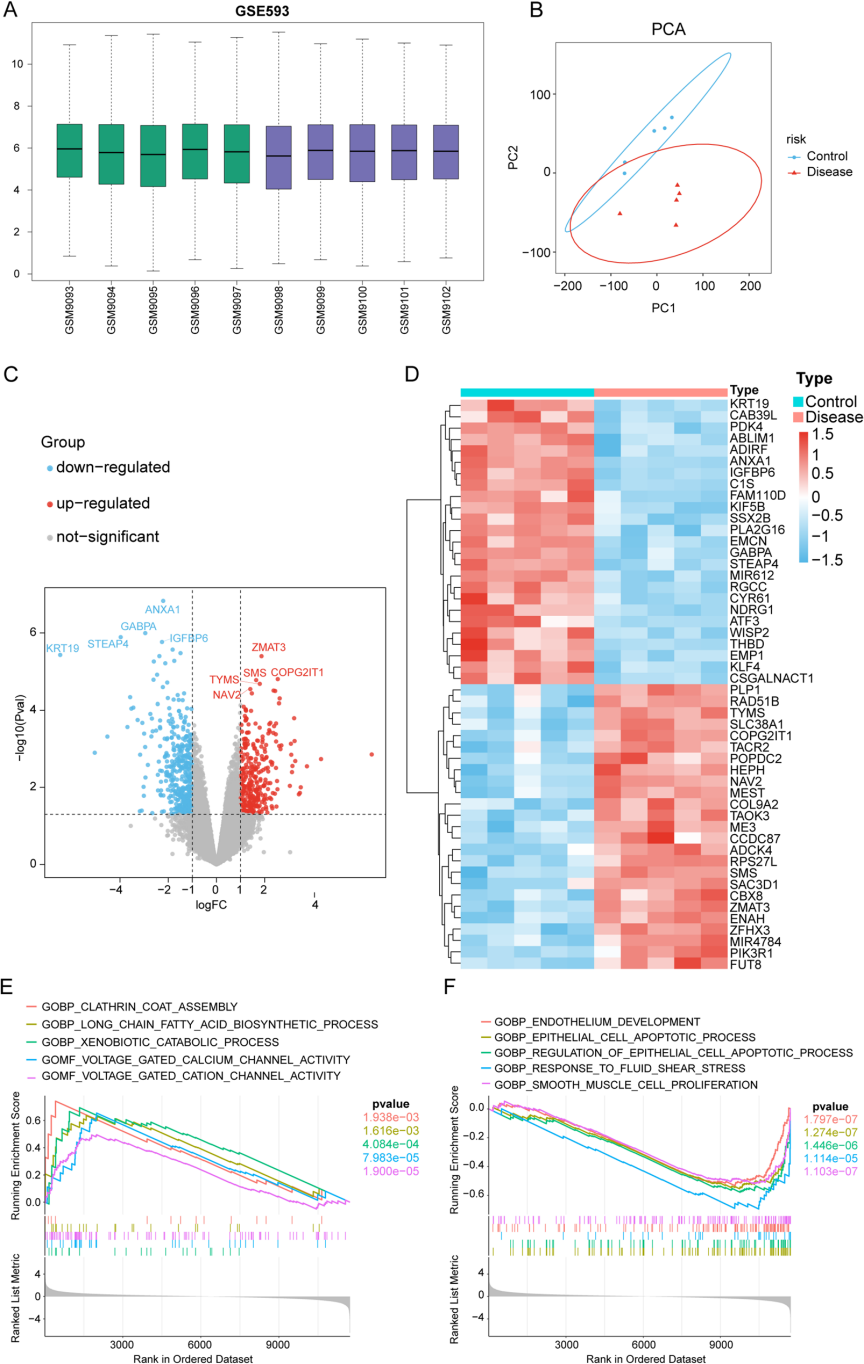
Differential gene analysis of the GSE593 dataset. **A** Box plot of the normalized samples. **B** Principal component analysis of the normal myometrial and uterine leiomyoma (control and disease group) tissues. **C** Volcano plot of the differentially expressed genes. Red indicates significant upregulation, whereas blue indicates significant downregulation in the disease group. **D** Cluster heatmap of the differentially expressed genes. **E** Gene set enrichment analysis (GSEA) of the top five signaling pathways most significantly upregulated in Gene Ontology (GO) functional enrichment analysis. **F** GSEA of the top five signaling pathways most significantly downregulated in GO functional enrichment analysis

To explore the potential therapeutic targets for uterine fibroids, we analyzed the GSE593 dataset, including the uterine leiomyoma (disease group; *n* = 5) and normal myometrial tissue (control group; *n* = 5) samples. Box plot of gene expression after sample normalization is shown in Fig. 1A. Horizontal axis indicates the samples sorted by group, and the vertical axis indicates the gene expression level. Expression distribution was relatively uniform in both the control (green) and disease (blue) groups. Principal component analysis results are shown in Fig. 1B. Sample points of the control and disease groups overlapped to a small extent; however, most areas were well-separated, indicating significant transcriptomic differences between the two groups. Differential expression analysis results are shown as a volcano plot (Fig. 1C and Supplementary Tabel 4). Red dots indicate the significantly upregulated genes, blue dots indicate the significantly downregulated genes, and gray dots indicate the genes with no significant expression differences. Top 5 most significantly upregulated and downregulated genes were noted (ranked by adjusted *p*-value). In total, 321 significantly upregulated and 412 significantly downregulated genes were identified. Based on the differential expression ranking, top 50 differential genes were selected to construct a heat map (Fig. 1D). Gene ontology functional enrichment analysis of DEGs was also performed. Top five upregulated and downregulated pathways with the most significant enrichment (ranked by false discovery rate *q*-value) were identified via gene set enrichment analysis (Fig. 1E and F).

### Screening for mitochondria-related DEGs

To identify the core genes closely associated with mitochondrial dysfunction in uterine fibroids, we performed WGCNA using the mitochondrial activity score (or other specifically defined scores) as the key phenotypic feature.

**Fig. 2:**
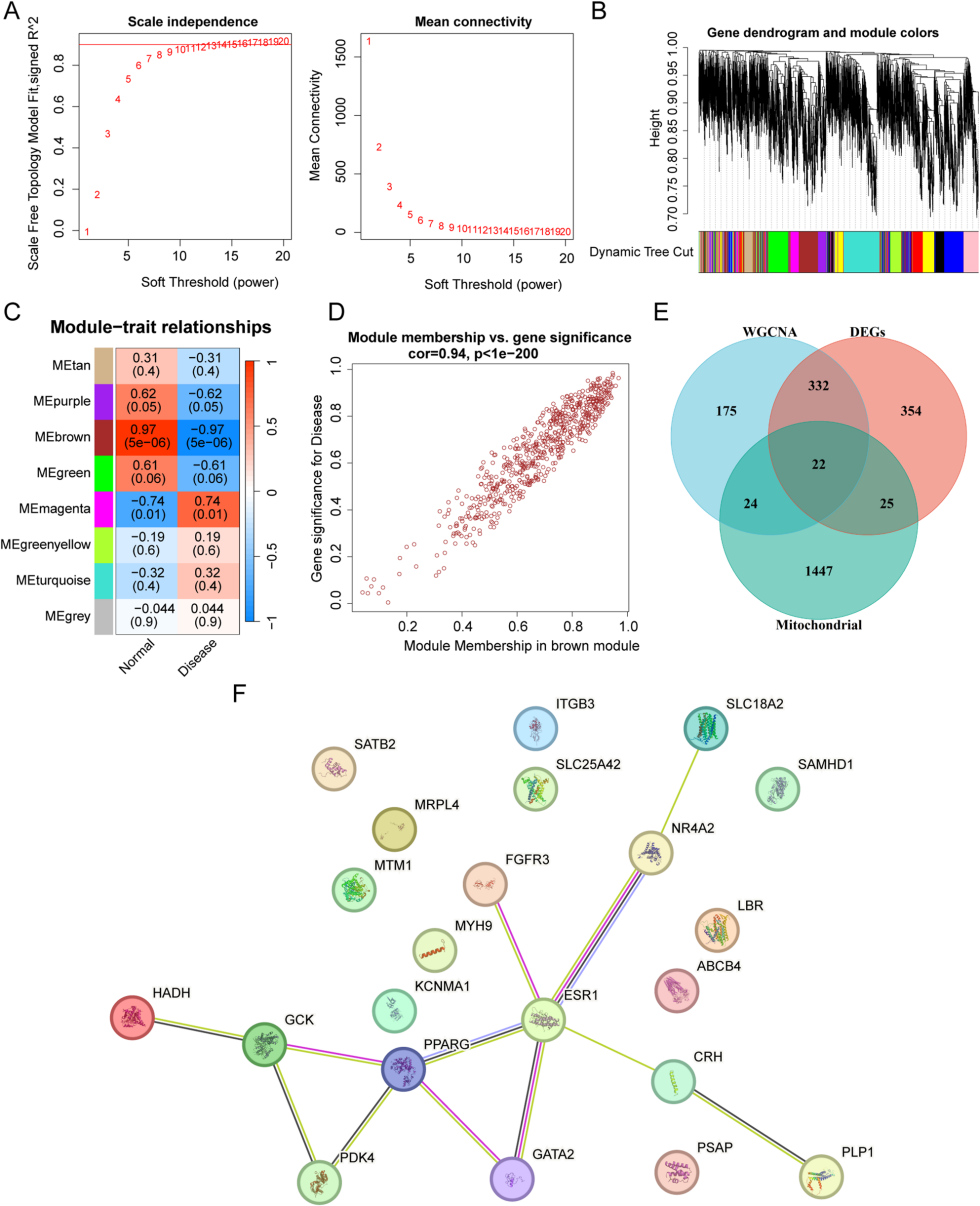
Screening for mitochondria-related differentially expressed genes. **A** Optimal soft threshold screening. **B** Dendrogram of hierarchical clustering with highly expressed genes. Similar modules were detected and combined. **C** Heatmap of the correlations between modules and traits. **D** Scatter plot of the brown module. **E** Venn diagram showing the intersection of differentially expressed genes from the weighted gene co-expression network analysis (WGCNA), GSE593, and mitochondria-related gene sets. **F** Twenty-two intersecting genes were imported into STRING 12.0 to construct a protein–protein interaction (PPI) network and identify the candidate genes (Proteins without known interactions under the selected confidence score are displayed as isolated nodes)

First, sample quality was assessed via hierarchical sample clustering. All samples met the analysis requirements, with no outliers detected. To construct the co-expression network, we evaluated the network topological properties under different soft-threshold powers. A power of 11 was selected for network construction. The scale-free topology fitting index (R²) was 0.9, indicating that the network possessed good scale-free properties (Fig. 2A). Dynamic tree-cutting algorithm was used to cluster the genes, and eight co-expression modules were identified. Module–trait correlation analysis revealed that the brown module exhibited the strongest positive correlation with the mitochondrial activity score (module eigengene correlation Cor = 0.94; *P* < 1.0 × 10⁻²⁰⁰; Fig. 2B and C). The brown module contained 553 genes (Fig. 2D).

To identify the mitochondria-related genes in the brown module, we obtained 1,518 mitochondria-related genes from the GeneCards database. The intersection of these mitochondrial, WGCNA brown module, and DEG sets was visualized using a Venn diagram (Fig. 2E). In total, 22 intersecting genes were obtained. To explore the interactions among the proteins encoded by these 22 genes, we constructed a PPI network using the STRING database (Fig. 2F). Through analysis of the network topological structure, we screened out hub genes with a degree centrality (degree) ≥ 3 as potential regulatory factors. Based on this criterion, we focused on three core hub genes, *ESR1* (degree = 5), *PPARG* (degree = 4), and *GCK* (degree = 3), for further in-depth analysis.

#### *ESR1* knockdown inhibits proliferation and induces ferroptosis in ELT3 cells

**Fig. 3:**
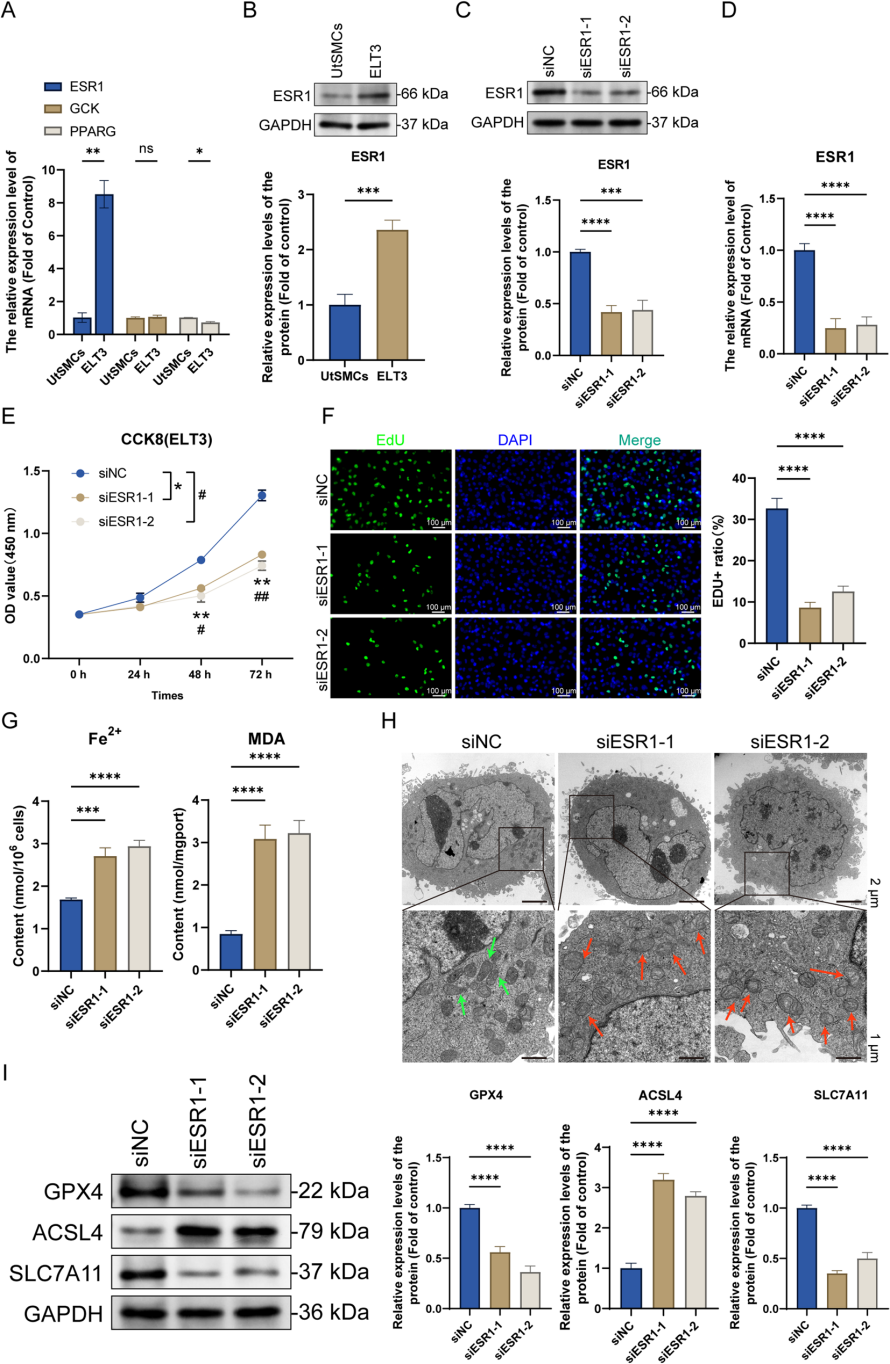
Estrogen receptor 1 (*ESR1*) knockdown inhibits proliferation and induces ferroptosis in ELT3 cells. **A** Relative mRNA expression levels of *ESR1*, peroxisome proliferator-activated receptor gamma (*PPARG*), and glycogen synthase kinase (*GSK*) in uterine smooth muscle cells (UtSMCs) and ELT3 cells were determined via reverse transcription-quantitative polymerase chain reaction (RT-qPCR) (mean ± SD, n = 3). **B** ESR1 protein levels in UtSMCs and ELT3 cells were determined via western blotting (mean ± SD, n = 3). **C** After transfecting the ESR1-interfering RNA into ELT3 cells and incubating for 48 h, relative ESR1 protein levels were determined via western blotting (mean ± SD, n = 3). **D** After transfecting the ESR1-interfering RNA into ELT3 cells and incubating for 48 h, relative *ESR1* mRNA levels were determined via RT-qPCR (mean ± SD, n = 3). **E** After transfecting the ESR1-interfering RNA into ELT3 cells and incubating for 48 h, cell viability was assessed via cell counting kit (CCK)-8 assay (mean ± SD, n = 3, *P < 0.05, siESR1-1 vs. siNC; ^#^*P* < 0.05, siESR1-2 vs. siNC). **F** After transfecting the ESR1-interfering RNA into ELT3 cells and incubating for 48 h, cell proliferation was assessed via 5-ethynyl-2’-deoxyuridine (EdU) assay (×200, scale bar = 100 μm) (mean ± SD, *n* = 3). **G** After transfecting the ESR1-interfering RNA into ELT3 cells and incubating for 48 h, Fe²⁺ and malondialdehyde (MDA) levels in the cell lysates were determined using the Fe²⁺ and MDA detection kits, respectively (mean ± SD, *n* = 3). **H** After transfection, mitochondrial morphological changes in ELT3 cells of each group were analyzed via transmission electron microscopy (Green arrow: Normal mitochondrial morphology; Red arrow: Damaged mitochondrial morphology, ×1500, scale bar = 2 μm; ×5000, scale bar = 2 μm) (mean ± SD, *n* = 3). **I** An ESR1 knockdown model was established in ELT3 cells, and the relative expression levels of GPX4, ACSL4, and SLC7A11 proteins were detected through Western blot experiments (mean ± SD, *n* = 3).^*^P < 0.05, ^**^P < 0.01, ^***^P < 0.001, and ^****^P < 0.0001; ns, *P* > 0.05

To confirm the bioinformatics analysis results, we measured the relative mRNA expression levels of *ESR1*, *PPARG*, and *GCK* in ELT3 cells and UtSMCs via RT-qPCR. Compared with UtSMCs, the expression level of ESR1 in ELT3 cells was significantly upregulated (*P* < 0.01), and the expression level of PPAGRG was significantly downregulated (*P* < 0.05), while the expression change of GCK was not statistically significant (Fig. 3A). Based on the RT-qPCR results and highest number of ESR1 nodes in the constructed bioinformatics-based PPI network, we selected *ESR1* for subsequent analyses. Western blotting confirmed the high ESR1 protein levels in UtSMCs (Fig. 3B). To verify the effects of ESR1 on ELT3 cells, we constructed an *ELT3* cell knockdown model via siRNA transfection. Western blotting and RT-qPCR confirmed the successful construction of the *ESR1* knockdown ELT3 cell model (Fig. 3C and D). Next, we assessed the effects of ESR1 on cell proliferation and ferroptosis in ELT3 cells. CCK8 and EdU assays showed that *ESR1* knockdown significantly reduced the ELT3 cell proliferation (Fig. 3E and F). Fe²⁺ catalyzes lipid peroxidation reactions to trigger ferroptosis, and MDA is the main toxic end-product and biomarker of lipid peroxidation [19]. In this study, *ESR1* knockdown significantly increased the Fe²⁺ and MDA levels in the cells (Fig. 3G), suggesting that ELT3 cells undergo ferroptosis. Mitochondria shrink, membrane density increases, cristae decrease in number or disappear, outer membrane remains intact, and the interior becomes densified, presenting a shrunken and condensed state in cells undergoing ferroptosis [20]. Transmission electron microscopy results indicated that in the siNC group, the cellular mitochondria exhibited normal morphological characteristics. There was no sign of mitochondrial swelling, the outer membrane remained continuous without dissolution, and the inner membrane cristae were clearly visible. In contrast, after ESR1 was knocked down, extensive abnormal changes occurred in the mitochondria, manifested as significant swelling. The integrity of the outer membrane was compromised, with dissolution occurring, and the inner membrane cristae also dissolved (see Fig. 3H). This series of results fully demonstrate that knocking down ESR1 can effectively induce mitochondrial damage. Since mitochondrial damage is one of the typical characteristics of ferroptosis, it further supports the relevant research conclusions. We detected the marker proteins related to ferroptosis. The results showed that, compared with the control group, after ESR1 knockdown, the expression level of GPX4 and SLC7A11 proteins decreased significantly, while the expression levels of ACSL4 protein increased significantly (Fig. 3I).

#### *ESR1* knockdown inhibits mitochondrial energy metabolism in ELT3 cells

**Fig. 4:**
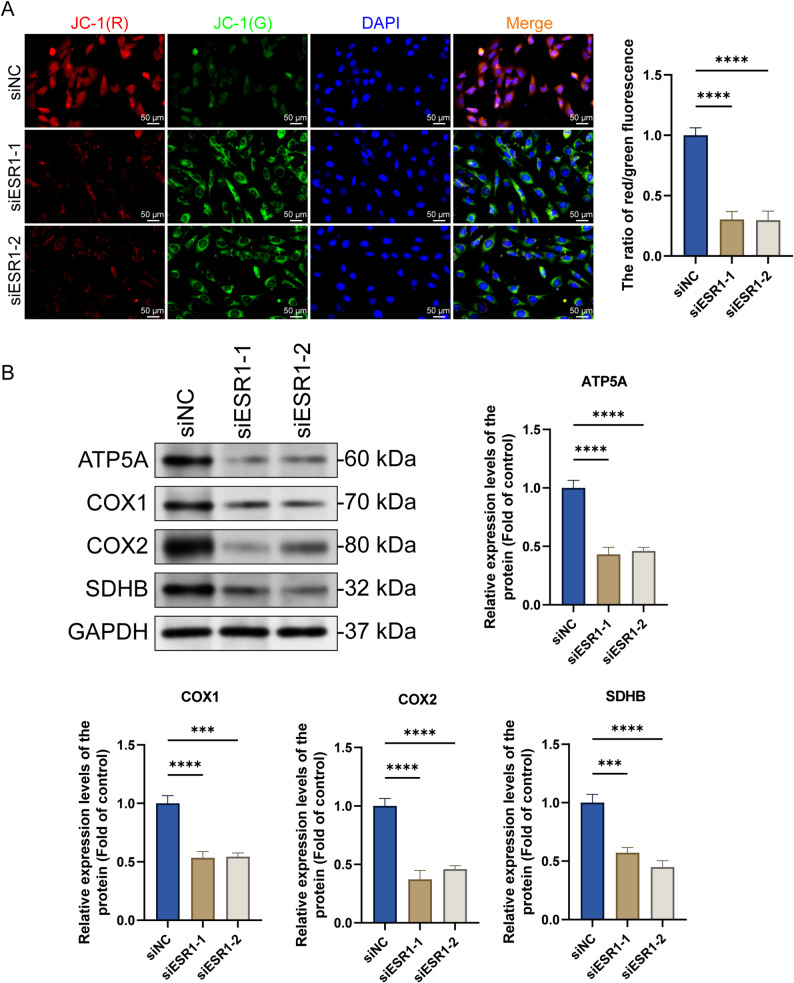
*ESR1* knockdown inhibits mitochondrial energy metabolism in ELT3 cells. **A** After transfecting the ESR1-interfering RNA into ELT3 cells and incubating for 48 h, mitochondrial membrane potential changes were detected using the 5,5’,6,6’-tetrachloro-1,1’,3,3’-tetraethylbenzimidazolcarbocyanine iodide (JC-1) fluorescent probe (×400, scale bar = 50 μm) (mean ± SD, *n* = 3). **B** After transfecting the ESR1-interfering RNA into ELT3 cells and incubating for 48 h, expression changes in the mitochondrial metabolism-related proteins, ATP5A1, COX1/2, and succinate dehydrogenase complex iron sulfur subunit B (SDHB), were assessed via western blotting (mean ± SD, *n* = 3). ^***^P < 0.001 and ^****^P < 0.0001

After determining that it causes mitochondrial structural damage (shrinkage in volume and disappearance of cristae), typical morphological feature of ferroptosis, we hypothesized that *ESR1* knockdown simultaneously impairs the mitochondrial functions, especially energy metabolism. As mitochondrial cristae are key OXPHOS sites, collapse of their structure inevitably affects the electron transport chain (ETC) activity and ability to synthesize ATP. Moreover, energy metabolism disorder is a driving factor of ferroptosis [21]. Therefore, we assessed the mitochondrial energy metabolism to clarify whether ESR1 deficiency induces ferroptosis by disrupting energy homeostasis. We detected changes in the mitochondrial membrane potential using the JC-1 fluorescent probe. *ESR1* knockdown significantly decreased the mitochondrial membrane potential, indicating disrupted mitochondrial functions (Fig. 4A). To confirm our hypothesis, we evaluated the mitochondrial energy metabolism-related protein levels. Western blotting showed that *ESR1* knockdown significantly decreased the ATP5A, COX1/2, and succinate dehydrogenase complex iron sulfur subunit B (SDHB) protein levels (Fig. 4B).

#### *ESR1* knockdown inhibits the Wnt/β-catenin signaling pathway

**Fig. 5:**
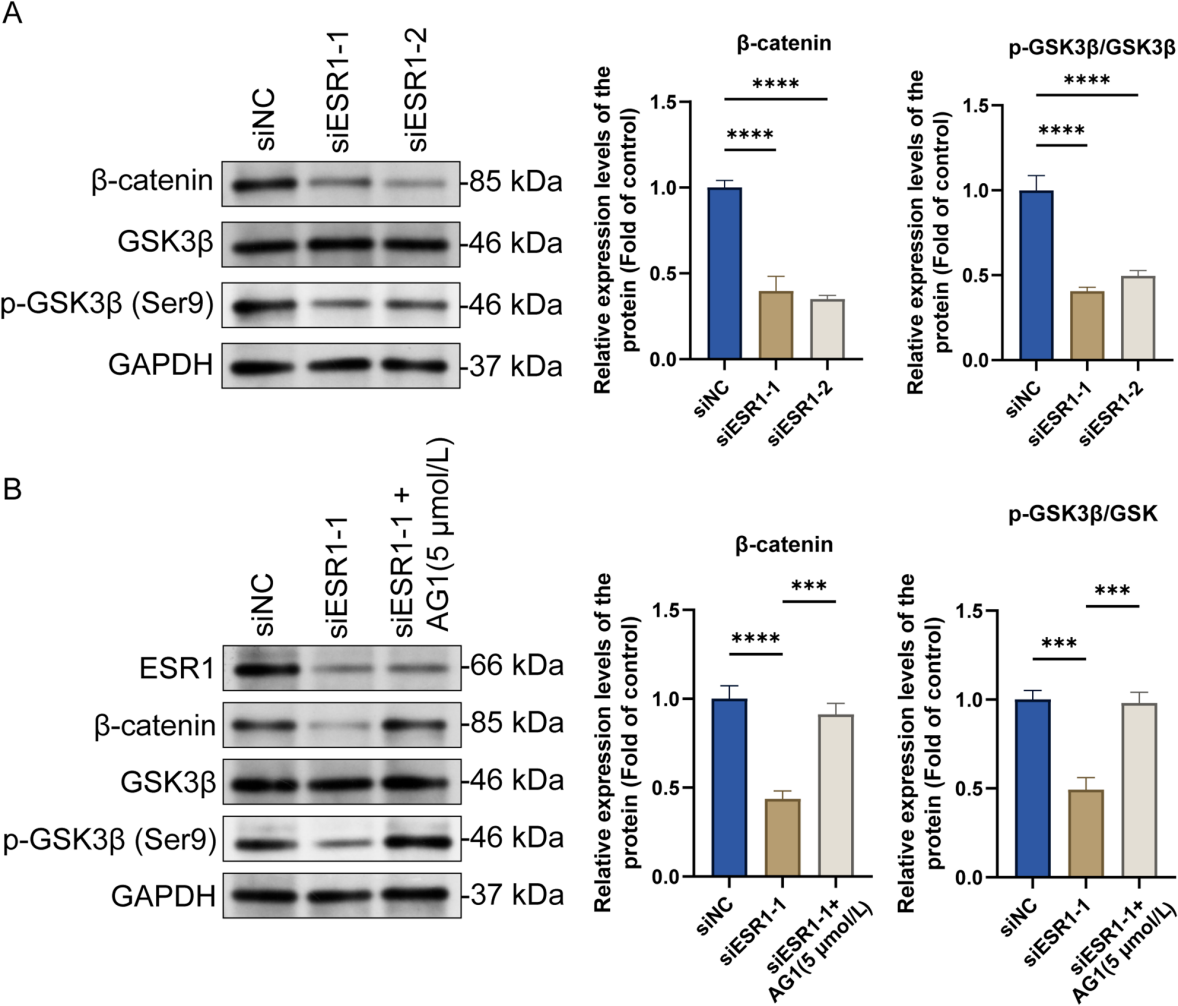
*ESR1* knockdown inhibits the Wnt/β-catenin signaling pathway. **A** After transfecting the ESR1-interfering RNA into ELT3 cells and incubating for 48 h, expression levels of key proteins in the Wnt/β-catenin signaling pathway, including β-catenin, p-GSK3β, and GSK3β, were determined via western blotting (mean ± SD, *n* = 3). **B** After transfecting the ESR1-interfering RNA into ELT3 cells and incubating for 24 h, AG1 (Wnt/β-catenin signaling pathway activator) was added, and the cells were incubated for another 48 h (mean ± SD, *n* = 3). Then, expression levels of ESR1 and key proteins in the Wnt/β-catenin signaling pathway, including β-catenin, p-GSK3β, and GSK3β, were determined via western blotting. ^***^P < 0.001 and ^****^P < 0.0001

Next, we explored the possible mechanisms by which ESR1 regulates mitochondrial energy metabolism to induce ferroptosis, focusing on the Wnt/β-catenin signaling pathway. Notably, *ESR1* knockdown significantly decreased the β-catenin protein levels and reduced the p-GSK-3β (Ser9)/GSK3β ratio. These results confirmed that *ESR1* knockdown significantly inhibited the Wnt/β-catenin signaling pathway activity (Fig. 5A). To further verify that ESR1 targets and regulates the Wnt/β-catenin signaling pathway, we performed a reverse-verification experiment using AG1. Notably, AG1 reversed the inhibitory effect of *ESR1* knockdown on the Wnt/β-catenin signaling pathway, further confirming the targeted regulatory effect of ESR1 on this pathway (Fig. 5B).

### AG1 reverses the inhibitory effects of *ESR1* knockdown on proliferation and ferroptosis induction in ELT3 cells

**Fig. 6:**
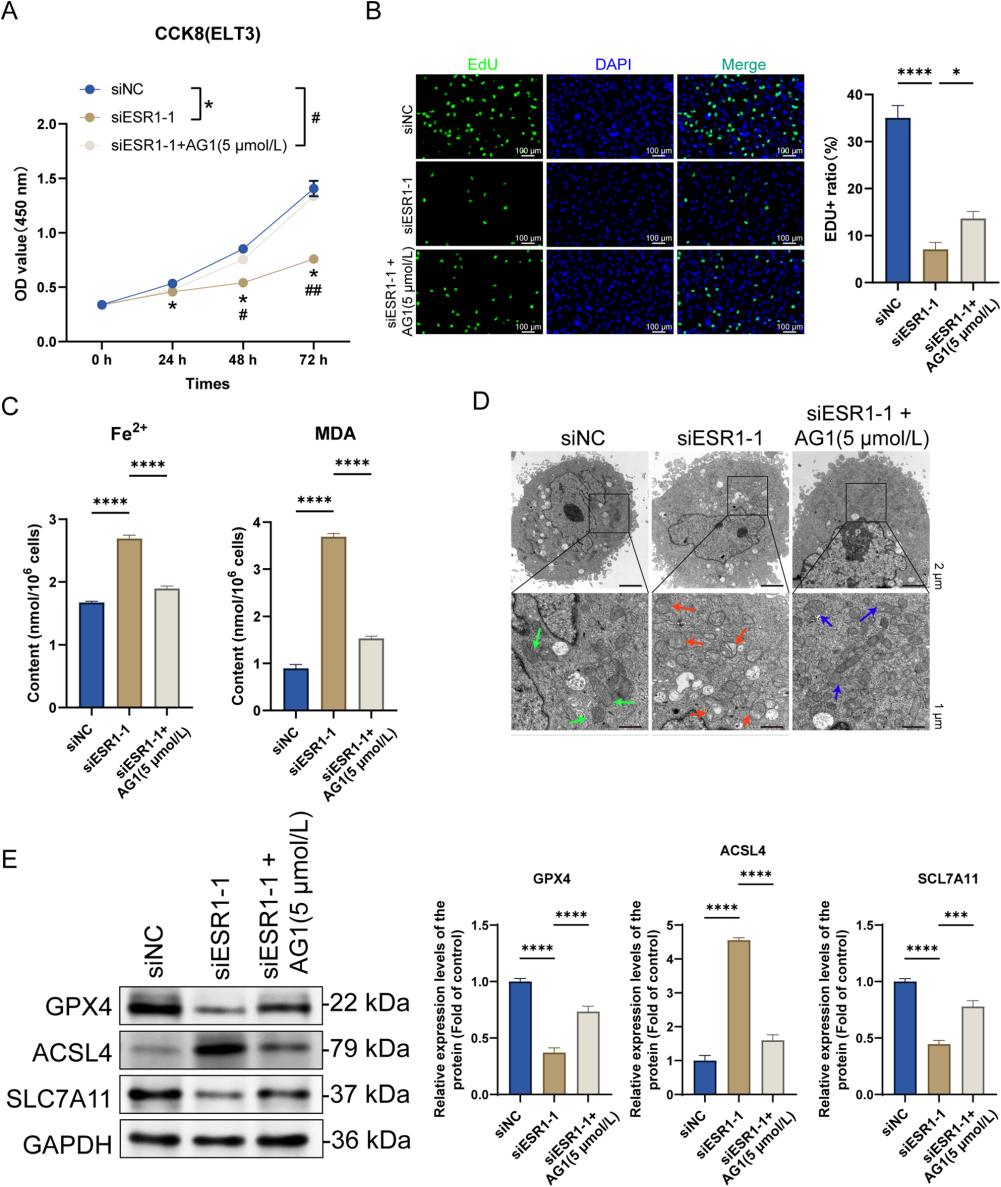
AG1 reverses the inhibitory effects of *ESR1* knockdown on proliferation and ferroptosis induction in ELT3 cells. **A** ELT3 cells were transfected with the ESR1-interfering RNA and incubated for 24 h. Then, AG1 (Wnt/β-catenin signaling pathway activator) was added, and the cells were incubated for another 48 h. Subsequently, ELT3 cell viability was assessed via CCK8 assay (mean ± SD, *n* = 3). **B** ELT3 cell proliferation in each group after treatment was assessed via EdU staining assay (×200, scale bar = 100 μm) (mean ± SD, *n* = 3). **C** Fe²⁺ and MDA levels in the ELT3 cells of each group were determined using the Fe²⁺ and MDA detection kits, respectively (mean ± SD, *n* = 3). **D** Changes in mitochondrial structure and morphology in the ELT cells of each group after treatment were assessed via transmission electron microscopy (Green arrow: Normal mitochondrial morphology; Red arrow: Damaged mitochondrial morphology; Blue arrow: Mitochondrial morphology after damage and repair, ×1500 and ×5000, scale bar = 2 μm) (mean ± SD, *n* = 3). **E** The changes in the relative expression levels of GPX4, ACSL4 and SLC7A11 proteins were detected by Western blot assay (mean ± SD, *n* = 3).^*^P < 0.05 and^****^P < 0.0001

After verifying *ESR1* knockdown inhibits the Wnt/β-catenin signaling pathway, we investigated whether the inhibitory effects of *ESR1* knockdown on proliferation and ferroptosis induction in ELT3 cells are achieved by inhibiting the Wnt/β-catenin signaling pathway. Indeed, *ESR1* knockdown significantly inhibited cell proliferation (Fig. 6A and B), decreased the intracellular Fe²⁺ and MDA levels, and disrupted the mitochondrial structure and morphology in ELT3 cells. However, AG1 reversed the effects of *ESR1* knockdown on ELT3 cell proliferation and ferroptosis (Fig. 6C and D). We measured the expression levels of ferroptosis - related proteins. The results showed that ESR1 knockout significantly reduced the relative expression levels of GPX4 and SLC7A11, and increased the relative expression level of ACSL4. After adding AG1, compared with the ESR1 - knockdown group, the expression level of GPX4 and SLC7A11 proteins increased significantly, while the relative expression levels of ACSL4 protein decreased significantly. (Fig. 6E). These results suggest that *ESR1* knockdown inhibits proliferation and induces ferroptosis in ELT3 cells by inhibiting the Wnt/β-catenin signaling pathway.

### AG1 reverses the inhibitory effect of *ESR1* knockdown on mitochondrial energy metabolism

**Fig. 7:**
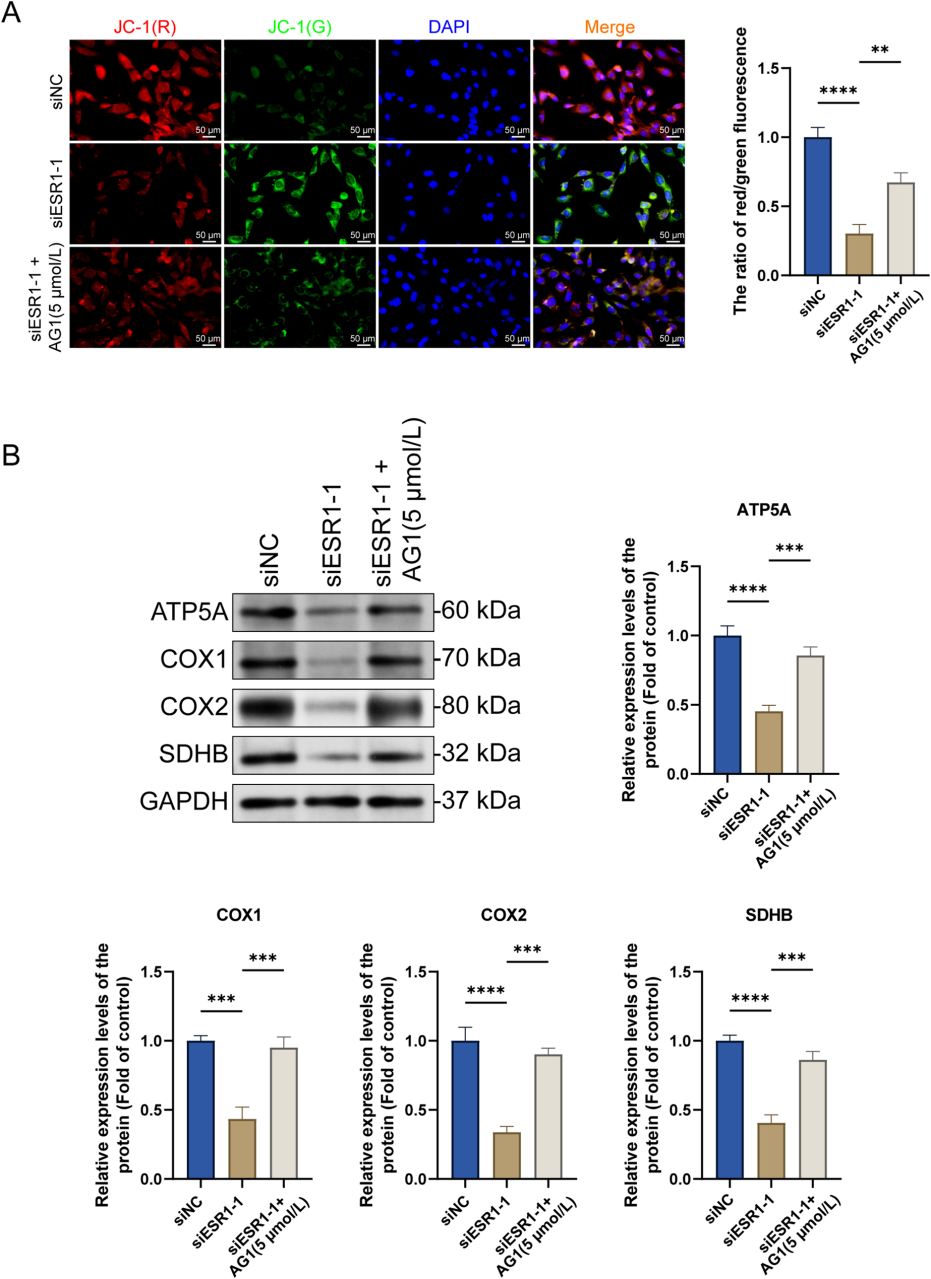
AG1 reverses the inhibitory effect of *ESR1* knockdown on mitochondrial energy metabolism. (A) ELT3 cells were transfected with the ESR1-interfering RNA and incubated for 24 h. Then, AG1 (Wnt/β-catenin signaling pathway activator) was added, and the cells were incubated for 48 h. Subsequently, mitochondrial membrane potential of ELT cells was detected using the JC-1 fluorescent probe (×400, scale bar = 50 μm) (mean ± SD, *n* = 3). (B) ELT3 cells were transfected with the ESR1-interfering RNA and incubated for 24 h. Subsequently, AG1 (Wnt/β-catenin signaling pathway activator) was added, and the cells were incubated for 48 h. Expression changes in the mitochondrial metabolism-related proteins, ATP5A1, COX1/2, and SDHB, were determined via western blotting (mean ± SD, *n* = 3). ^**^P < 0.01, ^***^P < 0.001, and ^****^P < 0.0001

Next, we determined whether the effect of ESR1 on mitochondrial energy metabolism is also achieved by regulating the Wnt/β-catenin signaling pathway. *ESR1* knockdown significantly decreased the mitochondrial membrane potential and ATP5A, COX1/2, and SDHB protein levels in ELT3 cells. However, AG1 reversed the inhibitory effect of *ESR1* knockdown on mitochondrial energy metabolism (Fig. 7A and B). These results suggest that *ESR1* knockdown inhibits mitochondrial energy metabolism in ELT3 cells by suppressing the Wnt/β-catenin signaling pathway.

## Discussion

To the best of our knowledge, this study is the first to demonstrate the crucial role of ESR1 in uterine fibroid cell proliferation by regulating mitochondrial energy metabolism. Previous studies have mostly focused on the classical nuclear transcriptional function of ESR1, with only a few investigating its non-genomic effect on mitochondrial homeostasis. By integrating the GSE593 transcriptome data with the GeneCards data, we found that ESR1 was the most crucial node connecting the DEGs and mitochondrial function network (with the highest degree value in the PPI network). OXPHOS is a core process in mitochondrial energy metabolism. SDHB is a key subunit of the mitochondrial ETC complex II [22]. COX1/2 are the core catalytic subunits of the terminal ETC complex IV, transferring electrons to the oxygen molecules (reducing them to water) and pumping protons to establish a proton gradient across the inner mitochondrial membrane [23]. ATP5A is the core catalytic subunit of ATP synthase (complex V) directly using the energy released by the reflux of the proton gradient to catalyze ATP synthesis. Therefore, SDHB, COX1/2, and ATP5A are functionally closely linked; SDHB participates in early electron transfer, COX1/2 completes terminal electron transfer and maintains the proton gradient, and ATP5A synthesizes ATP using the proton gradient [24]. Together, they determine the mitochondrial OXPHOS and cellular energy production efficiencies. In our functional experiments, *ESR1* knockdown collapsed the mitochondrial cristae structure, decreased the membrane potential, and significantly decreased the ATP5A, COX1/2, and SDHB levels, suggesting impaired OXPHOS in cells. The ability of ESR1 to regulate mitochondrial functions and metabolism has been demonstrated in other diseases and conditions, such as type II diabetes and heart disease [7, 8], supporting the reliability of our study results. However, this study is the first to demonstrate the effects of ESR1 on uterine fibroid cells. The tested cells exhibited the typical features of ferroptosis: Increased Fe²⁺ and MDA levels, mitochondrial shrinkage, and cristae disappearance. Our results suggest that ESR1 inhibition induces ferroptosis in uterine leiomyoma tissues. Ferroptosis is an iron-dependent form of cell death characterized by the accumulation of lipid peroxides. In recent years, its role in the occurrence, development, and treatment of gynecological diseases has attracted significant attention [25]. We focused on three key regulatory proteins. GPX4, a core antioxidant enzyme, can inhibit ferroptosis [26]. ACSL4, on the other hand, promotes the production of peroxidation-prone lipids, thus accelerating the process [27]. SLC7A11 is responsible for synthesizing the precursor of glutathione, providing support for GPX4 [28]. The results of this study indicate that knocking out ESR1 reduces the levels of GPX4 and SLC7A11, while increasing the level of ACSL4. This combination of protein expression changes (weakening of the key defense mechanism and enhancement of pro-death signals) collectively indicates that ESR1 deficiency promotes ferroptosis. Notably, the addition of AG1 was able to reverse the changes in all these proteins, demonstrating that the Wnt/β-catenin signaling pathway may be involved. *ESR1* knockdown also induces ferroptosis in breast cancer cells [29]. Additionally, ESR1 serves as a characteristic liver fibrosis marker [30]. Therefore, ESR1 regulates cellular ferroptosis in other diseases, consistent with our results.

Mechanistically, upon Wnt pathway activation, Ser9 site of GSK3β is phosphorylated to form p-GSK3β (Ser9). This modification inhibits the GSK3β kinase activity and prevents β-catenin degradation. Consequently, β-catenin accumulates in the cytoplasm and translocates into the nucleus to activate the target genes. Therefore, increased p-GSK3β (Ser9) activity is a marker of Wnt pathway activation. It stabilizes β-catenin by inactivating GSK3β and drives signal transduction. This study revealed the close association between ESR1 and the Wnt/β-catenin pathway. *ESR1* silencing downregulated the total β-catenin levels and p-GSK3β (Ser9)/GSK3β ratio, indicating accelerated β-catenin degradation. However, AG1 reversed these changes and restored the mitochondrial respiratory chain protein levels and cell proliferation, indicating that β-catenin is a key effector molecule for ESR1 to regulate the downstream metabolic and proliferative events. A previous study demonstrated the potential regulatory relationship between ESR1 and Wnt/β-catenin [9]. Inhibition of the Wnt/β-catenin pathway inhibits uterine leiomyoma cell proliferation [31], consistent with the results of this study. Currently, some drug treatments for uterine fibroids, such as GnRH agonists, mainly cause the shrinkage of fibroids by creating a hypoestrogenic state. However, their side effects, such as menopausal symptoms and bone loss, limit long-term use [32, 33]. Our study for the first time reveals a novel mechanism by which ESR1 regulates mitochondrial energy metabolism and cellular ferroptosis through the Wnt/β-catenin pathway. This provides a strong theoretical basis for the development of “targeted metabolism” therapies for uterine fibroids. For example, directly targeting ESR1 in fibroid cells or key proteins in mitochondrial OXPHOS downstream of it (such as ATP5A, COX1/2) may be able to more precisely induce the death of fibroid cells (such as ferroptosis), while avoiding systemic hypoestrogenic side effects. In addition, the key molecules in the ESR1-β-catenin-mitochondrial function axis we discovered (such as p-GSK3β Ser9) may serve as novel biomarkers for evaluating disease activity or treatment response.

Despite the encouraging results, this study has some limitations. First, this study did not perform clinical sample verification of the ESR1 levels and Wnt/β-catenin signaling pathway activation status in the uterine leiomyoma and normal smooth muscle tissues. Therefore, future research should utilize clinical samples to validate our conclusions. Secondly, this study mainly involves in-vitro cell experiments and lacks in - vivo animal experiments. It is necessary to introduce the use of β-catenin siRNA or GSK3β inhibitors in both in-vitro and in-vivo experiments to accurately evaluate the impact of ESR1 on the Wnt/β-catenin signaling pathway in uterine leiomyomas. Third, although this study preliminarily confirmed that ESR1 induces ferroptosis and disrupts mitochondrial energy metabolism in uterine leiomyoma cells, the causal relationship between mitochondrial energy metabolism and ferroptosis remains unclear. Therefore, reverse verification experiments, such as mitochondrial function rescue experiments, are necessary to determine the mechanistic link between mitochondrial energy metabolism and ferroptosis in uterine leiomyomas.

## Conclusions

Overall, ESR1 enhanced mitochondrial oxidative phosphorylation by activating the Wnt/β-catenin signaling pathway, driving uterine leiomyoma cell proliferation. However, our findings suggest that its knockdown induces ferroptosis in leiomyoma cells, revealing a new strategy to prevent uterine leiomyoma recurrence.

## Supplementary Information

Supplementary Material 1. Supplementary Tabel 1: ESR1 siRNA interference sequences and NC sequences.

Supplementary Material 2. Supplementary Tabel 2: Primer sequences for RT-qPCR.

Supplementary Material 3. Supplementary Tabel 3: Antibody information and dilution ratios for western blot experiments.

Supplementary Material 4. Supplementary Tabel 4: Summary Table of differentially expressed genes in GSE593 dataset.

Supplementary Material 5. Western blot raw images.

## Data Availability

The datasets used and/or analyzed in this study are available from the corresponding author upon reasonable request.

## Abbreviations

CCK-8: Cell counting kit-8
DEG: Differentially expressed gene
EdU: 5-ethynyl-2’-deoxyuridine
ESR1: Estrogen receptor 1
GCK: lucokinase
GSK-3β: Glycogen synthase kinase-3β
JC-1: 5,5’,6,6’-tetrachloro-1,1’,3,3’-tetraethylbenzimidazolcarbocyanine iodide
MDA: Malondialdehyde
OXPHOS: Oxidative phosphorylation
PPARG: Peroxisome proliferator-activated receptor gamma
PPI: Protein–protein interaction
RT-qPCR: Reverse transcription-quantitative polymerase chain reaction
SDHB: Succinate dehydrogenase complex iron sulfur subunit B
UtSMCs: Uterine smooth muscle cells
WGCNA: Weighted gene co-expression network analysis
